# Non-neoplastic cholangiopathies: an algorithmic approach

**DOI:** 10.1590/0100-3984.2019.0069

**Published:** 2020

**Authors:** Marina Silva Zacarias, Hanna Rafaela Ferreira Dalla Pria, Rafael Andrade Santiago de Oliveira, Luis Fernando Delmonte, Fernanda Garozzo Velloni, Giuseppe D’Ippolito

**Affiliations:** 1 Departamento de Diagnóstico por Imagem - Escola Paulista de Medicina da Universidade Federal de São Paulo (EPM-Unifesp), São Paulo, SP, Brazil.

**Keywords:** Bile duct diseases, Liver cirrhosis, Cholangiography, Computed tomography, Magnetic resonance imaging, Doenças das vias biliares, Cirrose hepática, Colangiografia, Tomografia computadorizada, Ressonância magnética

## Abstract

Cholangiopathies are chronic diseases that affect the bile ducts, comprising a heterogeneous group of progressive and potentially fatal entities. The diagnosis of these diseases is a great challenge for radiologists because of the overlapping of their clinical, biochemical, and imaging findings. Nevertheless, identifying the precise etiology is crucial, given that the therapeutic options are distinct and influence the prognosis of the patient. The purpose of this review article is to discuss some of the non-neoplastic causes of cholangiopathies and to provide a useful diagnostic algorithm.

## Abstract (pt)

As colangiopatias são doenças crônicas que afetam as vias biliares, constituindo um grupo heterogêneo de doenças progressivas e potencialmente fatais. O seu diagnóstico configura um grande desafio para os radiologistas, uma vez que seus aspectos clínicos, laboratoriais e de imagem se sobrepõem. Apesar disso, a busca etiológica é crucial, tendo em vista que as opções terapêuticas são distintas e influenciam o prognóstico do paciente. O objetivo deste artigo de revisão é discutir algumas das causas não neoplásicas de colangiopatias e fornecer um algoritmo útil na abordagem diagnóstica.

## INTRODUCTION

Cholangiopathies are chronic diseases that affect the bile ducts, targeting cholangiocytes. In this article we address the non-neoplastic causes of this heterogeneous group of slowly progressing diseases that are potentially fatal and are the third leading cause of liver transplantation in the United States^(1,2)^. Despite their various etiologies, these diseases have similar signs and symptoms, usually manifesting as jaundice, pain, weight loss, and fever. Their imaging findings also often overlap, making it difficult to identify their cause^(3,4)^. To gain a better understanding of their different forms of presentation, it is important to know the anatomy of the bile ducts, as well as the main pathophysiological mechanisms involved.

The intrahepatic bile ducts are lined by a single layer of epithelial cells, known as small cholangiocytes. The cholangiocyte population gradually increases toward the extrahepatic bile ducts, where they are known as large cholangiocytes^(5,6)^. The peribiliary glands, a source of new cholangiocytes and progenitor cells, are located throughout the bile duct, from the septal ducts to the extrahepatic ducts^(1,5)^. The bile ducts are irrigated by branches of the hepatic artery, which form the peribiliary plexus, and are drained through portal branches and centrilobular veins^(6)^.

Cholangiocytes have various morphologies and play different roles depending on their location in the biliary tree. That is partly due to their embryologic development, the intrahepatic ducts originate from hepatoblasts and the extrahepatic ducts originate from pancreatic and duodenal endodermal cells^(5,6)^. This also affects the expression of membrane proteins, which ultimately changes the way cholangiocytes react to endogenous and exogenous stimuli^(1,6)^.

It is now widely known that cholangiocytes not only produce and transport bile, but also actively participate in repairing and remodeling the biliary epithelium; small cholangiocytes, in particular, maintain their totipotent characteristics and play a special role in the repair of liver cell damage^(1,5,6)^.

In general, the pathophysiological mechanism of cholangiopathies involves a primary insult to cholangiocytes, inducing apoptosis of part of them whereas the other part becomes either senescent or reactive^(6)^. Reactive cholangiocytes release pro-inflammatory cytokines, chemotactic cytokines, and growth factors, which, in turn, recruit inflammatory and mesenchymal cells and stimulate angiogenesis. An imbalance among these responses leads to periportal fibrosis, ductopenia, and, finally, biliary cirrhosis^(1,5,6)^. Senescent cholangiocytes can remain in an inert state or become hypersecretors of pro-inflammatory cytokines, which also leads to an inflammatory response and fibrosis^(6)^. These last mentioned cholangiocytes have an even greater susceptibility to malignancy and are more commonly linked to the development of cholangiocarcinomas^(6)^.

Because cholangiopathies represent a heterogeneous group of diseases, a classification system has recently been proposed in an attempt to group them by etiological category^(6)^-congenital, immune-mediated, infectious, malignant, idiopathic, and other-as detailed in Table 1. Below, we will discuss some non-neoplastic causes of cholangiopathies, including iatrogenic cholangiopathy, and provide a useful diagnostic algorithm to help in their differentiation, using a combination of ultrasound, computed tomography (CT), and magnetic resonance imaging (MRI) findings, as well as epidemiological, clinical, and biochemical characteristics.

**Table 1:** Classification of cholangiopathies.

Congenital
Alagille’s syndrome
Caroli’s syndrome
Cystic fibrosis
MDR-3 deficiency
Polycystic liver disease (autosomal dominant polycystic liver disease, autosomal dominant polycystic kidney disease, autosomal recessive polycystic kidney disease)
Immune-mediated
Allograft rejection
Graft-versus-host disease
Primary biliary cholangitis
Idiopathic
Biliary atresia
Idiopathic childhood/adulthood ductopenia
IgG4-related cholangiopathy
Primary sclerosing cholangitis
Eosinophilic cholangitis
Sarcoidosis
Infectious
AIDS-related cholangiopathy
Bacterial cholangitis (caused by *Escherichia coli, Klebsiella spp., Enterococcus, Enterobacter, Pseudomonas spp.*, anaerobic microorganisms, etc.)
Parasitic cholangitis (caused by *Ascaris lumbricoides, Opisthorchis viverrini, Clonorchis sinensis, Fasciola hepatica*, etc.)
Malignant
Cholangiocarcinoma
Other
Drug-induced cholangiopathy (caused by the use of ketamine, 5-fluorouracil, etc.)
Vascular/ischemic cholangiopathy (e.g., post-transplant hepatic artery stenosis and systemic vasculitis)

*Source:* Table adapted from Cheung et al.^(6)^

## CONGENITAL CHOLANGIOPATHIES

### Caroli syndrome

Caroli syndrome is a rare autosomal recessive disease related to a malformation of the ductal plate that leads to a chronic inflammatory process and remodeling. When there is involvement of the large intrahepatic bile ducts, it is known as Caroli disease, which is characterized by fusiform and saccular dilatations that communicate with the rest of the biliary tract. When the disease occurs at the level of the interlobular ducts, it results in congenital hepatic fibrosis. Caroli syndrome results from the combination of these two conditions^(7,8)^. It can also be accompanied by kidney abnormalities, such as medullary sponge kidney and autosomal (dominant or recessive) polycystic kidney disease^(8)^. The symptoms are related to its complications, such as hepatolithiasis, cholangitis, liver abscess, and liver failure^(7,8)^.

**Figure 1:**
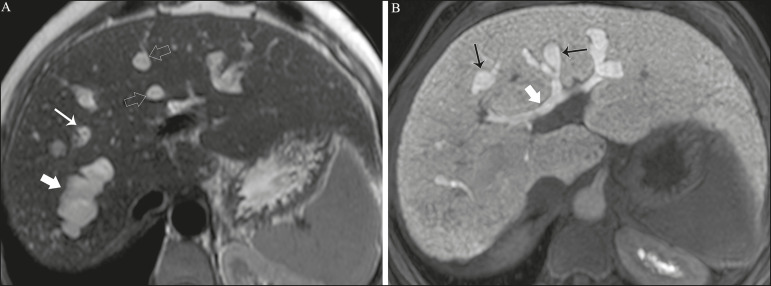
Axial T2-weighted MRI sequence (**A**) and axial T1-weighted MRI sequence in late hepatobiliary phase (**B**), showing dilatation of the intrahepatic bile duct (thick arrows) and sparse cysts (open arrows), some with a central dot of hypointense signal on T2 (thin white arrow), filled with the contrast agent in the late phase (black arrows), proving that there is communication with the bile duct, which is consistent with the diagnosis of Caroli disease.

In individuals with Caroli syndrome, imaging examinations show cystic dilatation of the intrahepatic bile ducts (most commonly in the right liver lobe), focal biliary stenoses, hepatolithiasis, and fibrosis. In some cases, fusiform dilatations of extrahepatic bile ducts may also be present as a result of stones passage and recurrent cholangitis. A hallmark of the disease is the central dot sign, which corresponds to the fibrovascular bundle in the center or in the periphery of a dilated bile duct^(7,8)^, as depicted in Figure 1. Magnetic resonance cholangiopancreatography (MRCP) can reveal signs that are very suggestive of this disease, such as those mentioned above. In more difficult cases, the workup can be complemented by an MRI scan with hepatobiliary-specific contrast agents (functional MRCP). In this diagnostic modality, it is possible to confirm whether the cysts communicate with the bile duct, because, if so, the contrast excreted by hepatocytes fills the bile duct and the cysts, what can be detected in delayed acquisitions-images acquired from 10 min to 24 h after contrast injection^(9,10)^-as shown in Figure 1.

Main differential diagnoses are primary sclerosing cholangitis and recurrent pyogenic cholangitis. These two conditions manifest as biliary dilatations, most often fusiform and sparse, alternating with segmental stenoses, and may be accompanied by stone, although primary sclerosing cholangitis is often accompanied by inflammatory bowel diseases. Saccular dilatations should be the focus of the investigation; if present, they favor the diagnosis of Caroli disease^(8)^.

### Congenital hepatic fibrosis

Congenital hepatic fibrosis is part of the group of diseases related to malformation of the ductal plate. It is an autosomal recessive condition and, like Caroli disease, it can be accompanied by autosomal dominant polycystic kidney disease, autosomal recessive polycystic kidney disease, and medullary sponge kidney^(11,12)^.

Congenital hepatic fibrosis affects the interlobular bile ducts and therefore does not manifest macroscopically with dilatation of the bile ducts, but as hepatic fibrosis and portal hypertension. When there is concomitant dilatation of the bile ducts, the association with other entities comprising the ductal plate malformation group, such as Caroli disease, biliary hamartomas, or choledochal cyst, must be considered^(11)^. Because it occurs in a chronic and insidious way, symptoms appear only in adolescence or early adulthood and are related to complications of portal hypertension^(11,12)^.

**Figure 2:**
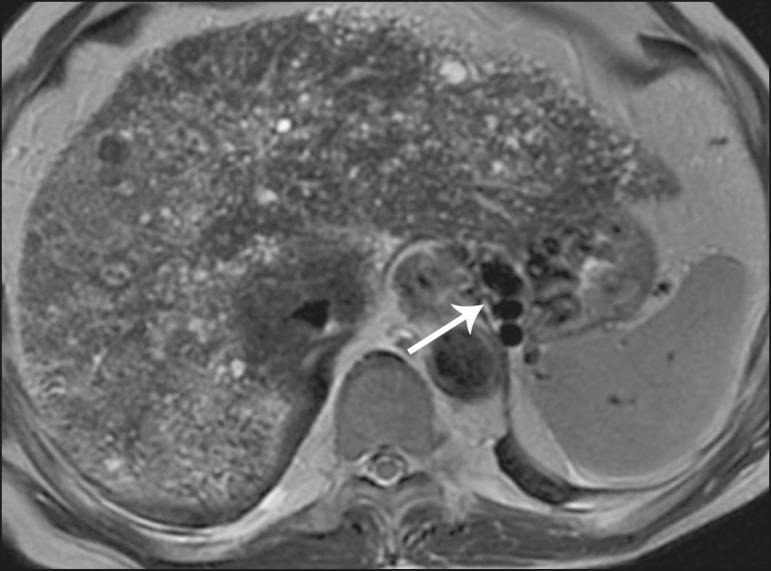
Numerous liver cysts and signs of portal hypertension with perigastric collaterals (arrow). A magnetic resonance elastography test (at 5.9 kPa) revealed fibrosis. Such findings are suggestive of congenital hepatic fibrosis.

Although the definitive diagnosis of congenital hepatic fibrosis is based on the histopathological findings, imaging examinations have been gaining importance. Approximately 80% of patients present at least three of the following^(12)^: morphological liver changes (hypertrophy of the medial segment of the left lobe, hypertrophy of the caudate lobe, and atrophy of the right lobe); splenomegaly; portosystemic collateral circulation; kidney abnormalities; and other ductal plate malformations, such as hamartomas and peribiliary cysts. Figure 2 shows some of the findings suggestive of congenital hepatic fibrosis.

### Polycystic liver disease

Polycystic liver disease is an autosomal dominant condition. It is part of the spectrum of congenital ductal plate malformations and affects the medium-caliber intrahepatic bile ducts. Therefore, it is often described as one of the cholangiopathies. During the involution of the ductal plate, the bile ducts lose communication with the normal biliary tract and dilate progressively throughout life, which is why the condition is also known as adult polycystic liver disease^(13,14)^. Two spectra of the disease are included in this category: autosomal dominant polycystic liver disease-in which there is involvement of the liver only-and autosomal dominant polycystic kidney disease-the most common form, which affects the kidneys and the liver concomitantly^(1,14)^. Patients are asymptomatic and only rarely experience symptoms related to hepatomegaly (e.g., pain, bloating, and dyspnea) or secondary to rupture, bleeding and infection of the cysts. Progression to liver failure is uncommon^(13,14)^.

In patients with polycystic liver disease, the only imaging findings are an enlarged liver, due to numerous peripheral or peribiliary cysts that do not communicate with the biliary tree, and that can sometimes show signs of bleeding or peripheral calcifications^(13)^. The size and number of cysts vary, with no clear cutoff values distinguishing between hereditary cystic disease (polycystic liver disease) and the nonhereditary form^(13,14)^.

### Cystic fibrosis

Hepatobiliary involvement occurs in up to 40% of patients with cystic fibrosis, liver cirrhosis being the second leading cause of death in patients with this disease^(15)^. The thick content and abnormal composition of the content in the bile ducts lead to a chronic inflammatory process, periportal fibrosis, and stones formation, which culminate with focal biliary fibrosis, stenoses, and dilatations of the intrahepatic and extrahepatic bile ducts, as well as gallbladder atrophy and parietal thickening, a hallmark of the disease. In rare cases, there can be progression to multilobular fibrosis and cirrhosis with portal hypertension^(16)^.

**Figure 3:**
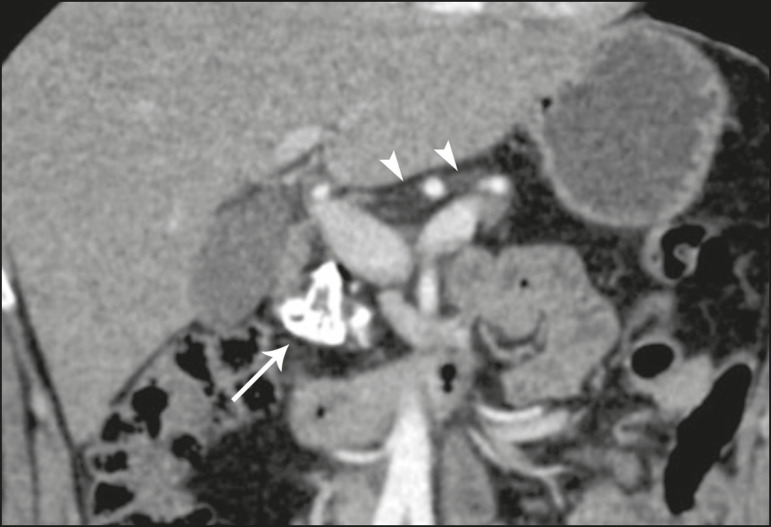
Patient with cystic fibrosis. Image shows atrophy and fatty replacement of the pancreas (arrowheads) with gross calcifications in the cephalic region (arrow).

In patients with cystic fibrosis, imaging examinations can reveal biliary duct abnormalities, which are seen in approximately 50% of patients, even in those without a known liver disease^(15)^. A common ultrasound finding is periportal hyperechogenicity, which on MRI is more often correlated with fat deposition than with fibrosis itself, having a positive correlation with the histopathological findings^(16)^. Another finding is gallbladder atrophy, which may be related to cystic duct stenosis or the presence of thick mucus^(15,16)^. Descriptions of the findings make it clear that these hepatobiliary manifestations overlap with those of primary sclerosing cholangitis, its main differential diagnosis^(16)^. In order to distinguish between the two, one should look for typical signs of cystic fibrosis in other organs (e.g., fatty replacement of the pancreatic parenchyma, pancreatic cysts, pancreatic calcifications, steatosis, and bronchiectasis)-as illustrated in Figure 3-and bear in mind that primary sclerosing cholangitis is usually accompanied by inflammatory bowel diseases^(16-18)^.

## IMMUNE-MEDIATED CHOLANGIOPATHIES

### Primary biliary cholangitis

Primary biliary cholangitis, formerly known as primary biliary cirrhosis, is an autoimmune disease of unknown cause that mainly affects women between the fifth and seventh decades of life. It is characterized by a chronic nonsuppurative inflammatory process of the interlobular bile ducts that results in fibrosis^(19,20)^.

In its early stages, primary biliary cholangitis is usually asymptomatic. However, in up to 50% of cases, it manifests as fatigue and severe pruritus, which may precede jaundice. It naturally progresses to cirrhosis, and the main effective clinical treatment is the use of ursodeoxycholic acid, although it is useful only in the early stages. When patients evolve to liver cirrhosis, the only curative treatment is liver transplantation^(19)^.

To establish a diagnosis of primary biliary cholangitis, at least two of the following criteria must be found^(19)^: elevated levels of cholestatic enzymes for over six months; presence of antimitochondrial antibodies; and liver biopsy findings consistent with the diagnosis (specifically nonsuppurative destructive cholangitis). The concomitant presence of other autoimmune diseases, such as Sjögren’s syndrome, is not uncommon^(21,22)^.

Because primary biliary cholangitis affects the interlobular bile ducts, which are microscopic structures, the imaging findings are subtle and often appear late (after the onset of cirrhosis). Usually, the biliary tree have a normal radiological appearance, but eventually there can be ductopenia, giving the aspect of “pruned tree” on MRCP images^(23)^.

**Figure 4:**
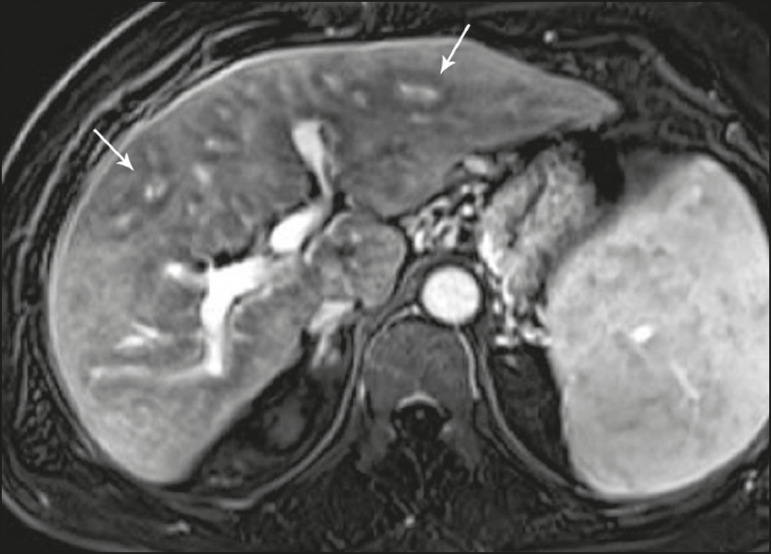
Periportal halo with a hypointense signal (arrows) in a patient with primary biliary cholangitis.

In recent studies, a periportal halo sign has been described on MRI images, consisting of the presence of a hypointense halo around the peripheral portal branches, which correlates with periportal fibrosis, best characterized in the portal and equilibrium phases (Figure 4). This finding is closely related to the stage of the disease, being more prominent in the more advanced stages^(19)^. Regional lymph node enlargement, notably in the hepatic hilum, is another common finding in patients with primary biliary cholangitis. Other findings are related to cirrhosis and portal hypertension, including the presence of regenerative nodular hyperplasia^(19,24)^.

## IDIOPATHIC CHOLANGIOPATHIES

### Primary sclerosing cholangitis

Primary sclerosing cholangitis is due to a chronic idiopathic inflammatory process of the intrahepatic and extrahepatic bile ducts. It is believed to be an autoimmune disease, mainly due to its strong association with inflammatory bowel diseases, being present in 60-80% of patients, particularly ulcerative colitis and, to a lesser extent, Crohn’s disease. It manifests between the fourth and fifth decades of life, with a predilection for men. The symptoms are nonspecific, including cholestatic syndrome and abdominal pain. The initial signs are often limited to elevated levels of cholestatic enzymes such as alkaline phosphatase and gamma-glutamyl transferase^(25)^.

Chronic involvement of the intrahepatic and extrahepatic bile ducts leads to progressive periductal fibrosis, which can evolve to liver cirrhosis. Many other sclerosing cholangitis conditions share those characteristics. Primary sclerosing cholangitis has no specific clinical, biochemical, imaging, or histopathological features, and it is diagnosed by ruling out secondary causes such as IgG4-related sclerosing cholangitis (IgG4-SC), acquired immunodeficiency syndrome related cholangiopathy, eosinophilic cholangitis, ischemic cholangitis, and recurrent pyogenic cholangitis^(17)^.

**Figure 5:**
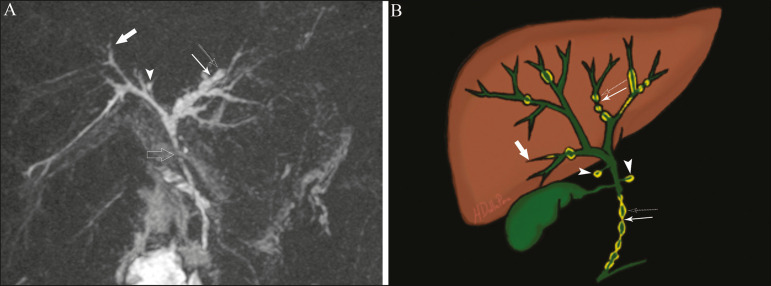
A 38-year-old male patient with ulcerative colitis and recurrent cholangitis. **A:** MRCP image showing stenosis of the common bile duct (open arrow) accompanied by a small area of irregular dilatation of the intrahepatic bile ducts and areas of stenosis (thin continuous arrow) alternated with dilated segments (dashed arrow), creating a beaded appearance; biliary diverticula (arrowhead); and obliteration of the peripheral bile ducts, creating a “pruned tree” appearance (thick arrow). The imaging findings and the clinical context suggest the diagnosis of primary sclerosing cholangitis. **B:** Schematic diagram.

In suspected cases of primary sclerosing cholangitis, the imaging examination of choice is MRCP, which can show multifocal involvement, usually affecting the intrahepatic and extrahepatic ducts concomitantly, with short, narrowed, irregular segments alternated with dilated segments or normal segments, giving it a beaded appearance. As the disease progresses, the peripheral ducts become obliterated and are no longer seen on MRI, creating a “pruned tree” appearance. Biliary diverticula can also be present, and a smaller percentage of patients (8%) have biliary stones^(17)^, as depicted in Figure 5. Chronic involvement causes significant morphological changes in the liver, such as atrophy of the lateral segments of the left lobe and of the posterior segments of the right lobe with compensatory hypertrophy of the caudate lobe, which may be accompanied by centrally distributed regenerative macronodules^(25)^.

Among patients with primary sclerosing cholangitis, 10-15% will develop cholangiocarcinoma, which is one of the most feared complications, occurring most commonly when the patient has both primary sclerosing cholangitis and an inflammatory bowel disease. One of the challenges is that early cholangiocarcinoma and primary sclerosing cholangitis share certain characteristics, such as luminal narrowing and wall thickening, which hinder their identification and can make the two entities indistinguishable in some cases^(17)^. The following features must be considered when trying to differentiate between them: extent of the stenotic segment; parietal thickness, hyperenhancement, and asymmetry; degree of luminal irregularity; and serum levels of the tumor marker CA 19-9, which are generally quite high in the presence of cholangiocarcinoma^(26-28)^.

It is important to note that there is no effective treatment for primary sclerosing cholangitis, liver transplantation being the only curative therapy, with a recurrence rate of up to 25% at 5-10 years after transplantation^(17)^.

### IgG4-related sclerosing cholangitis

The condition known as IgG4-SC is defined as biliary involvement in IgG4-related sclerosing disease. Because it is systemic, IgG4-SC can manifest in multiple organs. The biliary tract is one of the most common sites of involvement, second only after the pancreas, though there is concurrent involvement of both in most cases^(29)^. Approximately 90% of patients with IgG4-SC have autoimmune pancreatitis^(30)^. Other autoimmune diseases commonly seen in patients with IgG4-SC include nephritis, retroperitoneal fibrosis, sclerosing mesenteritis, and sialadenitis^(29)^.

The mean age of patients with IgG4-SC is 60 years, and most are men. Clinically, they present with cholestatic syndrome and symptoms related to autoimmune pancreatitis, such as weight loss, abdominal pain, and diabetes^(29)^. Laboratory tests show high serum levels of IgG4, a highly specific marker, and are usually positive for rheumatoid factor and antinuclear antibodies^(30)^. In some cases, the disease is self-limiting, whereas in others it progresses to cirrhosis. A hallmark of the disease is its excellent response to corticosteroids^(29)^.

**Figure 6:**
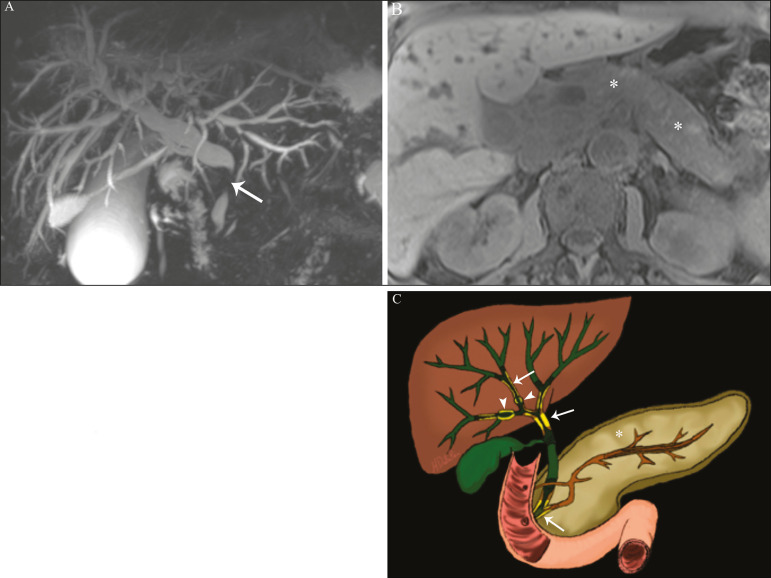
MRCP image (**A**) showing a long segment of stenosis in the common bile duct (arrow) with upstream dilatation of the biliary tree. **B**: Unenhanced axial T1-weighted MRI sequence showing pancreatic diffuse decreased signal intensity and loss of clefts definition (asterisks), suggestive of autoimmune pancreatitis. The clinical context and imaging findings suggest IgG4-related cholangiopathy. **C**: A schematic diagram showing prestenotic dilatation (arrowheads) of the intrahepatic bile ducts.

Although IgG4-SC affects the intrahepatic and extrahepatic bile ducts, the latter are more commonly involved, being affected in isolation in 43% of patients. On CT and MRI, the affected ducts show thickening, irregularity, and focal or diffuse contrast enhancement. The presence of prestenotic dilatation is another defining feature of the disease. The segments are generally long and continuous (Figure 6), with or without a surrounding tissue. In addition, there can be gallbladder wall thickening, which is hypoechoic on ultrasound and shows a hypointense signal on MRI^(29)^. These features make IgG4-SC a great mimic of cholangiocarcinoma and primary sclerosing cholangitis. Nevertheless, involvement of other organs, such as the pancreas and kidneys, and high serum levels of IgG4 (≥ 300 mg/dL) are distinguishing factors. When there is suspicion of primary sclerosing cholangitis, other characteristics may be useful, such as patient age (typically under 40 years), concomitant inflammatory bowel diseases, multifocal biliary involvement with narrowed segments followed by normal or dilated segments, and biliary diverticula^(29,30)^. Nevertheless, distinguishing between these diseases may not be possible, and in that case, a histopathological study is needed^(30)^.

### Eosinophilic cholangitis

Eosinophilic cholangitis is a rare disease of unknown cause, only a few cases having been described in the literature. It is characterized by eosinophilic infiltration in the biliary tract and fibrosis^(31)^. It may be accompanied by other diseases that are also characterized by eosinophilic infiltration, such as eosinophilic gastroenteritis, the defining characteristic of such diseases being peripheral eosinophilia, which is present in approximately 50% of patients. Patients with eosinophilic cholangitis usually have a good response to corticosteroid therapy^(31)^.

**Figure 7:**
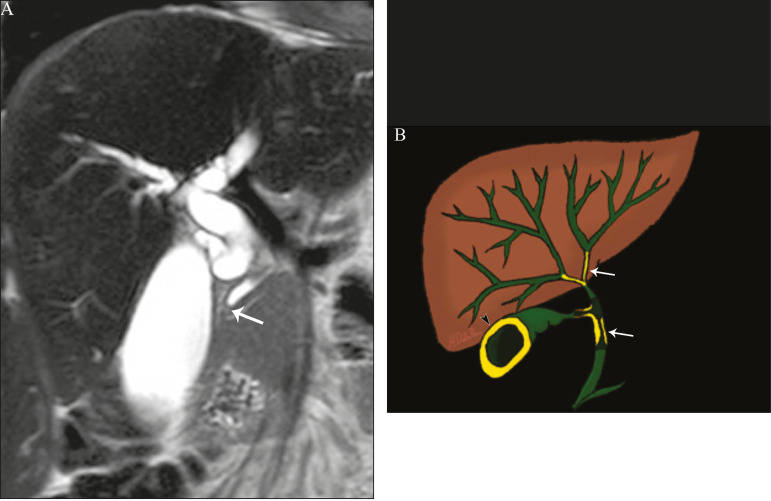
A 36-year-old male patient with a history of eosinophilic pneumonia who presented with abdominal pain, jaundice, and peripheral eosinophilia. **A**: Coronal T2-weighted MRI sequence showing abrupt narrowing of the distal intrapancreatic common bile duct (arrow) and upstream dilatation of the biliary tree. The pathology findings were consistent with eosinophilic cholangitis. **B**: A schematic diagram showing gallbladder wall thickening (arrowhead).

The imaging findings of eosinophilic cholangitis are nonspecific. There may be focal or diffuse involvement of the biliary tree, showing thickened walls, luminal narrowing, along with gallbladder wall thickening (Figure 7), the latter being a sign that can help when there is radiological suspicion of this disease^(17)^.

## INFECTIOUS CHOLANGIOPATHIES

### Recurrent pyogenic cholangitis

Recurrent pyogenic cholangitis is common in Asia, where infection with *Clonorchis sinensis*, a parasite associated with the disease, is endemic^(32)^. Other parasitic diseases related to recurrent pyogenic cholangitis are ascariasis and fascioliasis. It is believed that such parasitic infections can damage the ductal epithelium, particularly in the intrahepatic region, causing biliary dilatation without obstruction^(25)^. This can lead to bile stasis, luminal narrowing, and hepatolithiasis, which can also be caused by dietary factors or other agents, such as *Escherichia coli, Schistosoma mansoni*, and *Helicobacter pylori*. Structural abnormalities in the bile ducts, such as those seen in Caroli disease, ischemic cholangitis, and primary sclerosing cholangitis, are predisposing factors for recurrent pyogenic cholangitis. Although the symptoms are related to recurrent acute cholangitis, the disease can be asymptomatic, the only signs being abnormal laboratory test results, such as elevated levels of cholestatic enzymes^(17)^.

The pigmented calculi that form in the intrahepatic bile ducts of individuals with recurrent pyogenic cholangitis may or may not be accompanied by ductal narrowing. On CT scans, the stones are hyperdense; on MRI, they have an isointense signal in T2-weighted sequences and a hyperintense signal in T1-weighted sequences. The affected segment also presents parenchymal atrophy, usually occurring in the lateral segments of the left lobe and posterior segments of the right lobe, with hypertrophy of the other segments^(25)^. The risk of developing cholangiocarcinoma is also increased in patients with recurrent pyogenic cholangitis, occurring in up to 5%. Cholangiocarcinoma shows a predilection for atrophied segments and for segments with multiple stones^(32)^.

The main differential diagnosis of recurrent pyogenic cholangitis is primary sclerosing cholangitis. In recurrent pyogenic cholangitis, however, there is no beaded appearance or biliary diverticula, due to the fact that it usually affects only the intrahepatic region, as well as because it presents a disproportionate biliary dilatation, with peripheral distribution preferably^(17)^.

## VASCULAR CHOLANGIOPATHIES

### Ischemic cholangiopathy

As previously mentioned, the bile ducts are irrigated by the peribiliary plexus formed by branches of the hepatic artery and are therefore susceptible to reductions in the arterial blood supply. The most commonly affected segments are the middle third of the common bile duct and the confluence of the hepatic ducts, which can be affected focally or multifocally^(33)^.

**Figure 8:**
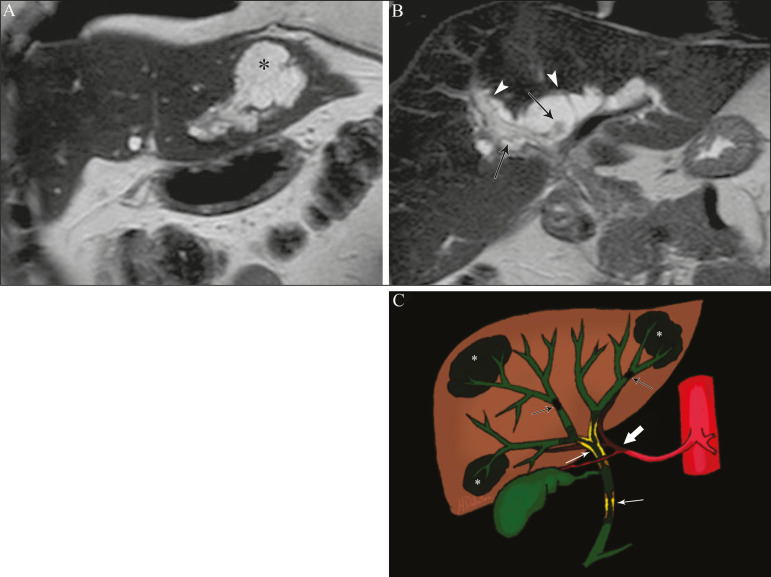
Patient presenting with thrombosis of the hepatic artery two years after liver transplantation. Coronal T2-weighted MRI sequences (**A** and **B**) showing severe dilatation of the biliary tract (arrowheads), hypointense content within the duct (black arrows), and a biloma (asterisk) contiguous to the dilated bile duct. The clinical context and the imaging findings suggest ischemic cholangiopathy. **C**: schematic diagram showing hepatic duct stenosis (thin white arrows) and hepatic artery thrombosis (thick arrow).

Ischemic lesions of the bile duct can be roughly divided into three types^(17)^: necrosis with biloma formation; epithelial desquamation with biliary obstruction; and fibrosis with ductal stenosis. Necrosis with biloma formation usually occurs as a consequence of severe arterial insufficiency, with necrosis throughout the duct wall, followed by parietal discontinuity and biloma formation (Figure 8). The bilomas can be seen on imaging as fluid collections adjacent to the affected ducts^(33)^. The second type mentioned above results from milder arterial insufficiency, with desquamation of the epithelium, which accumulates in the lumen of the duct and can lead to biliary obstruction. The desquamated epithelium forms biliary casts within the ducts, which, like stones, are seen on MRI as hyperintense areas on T1-weighted sequences and hypointense areas on T2-weighted sequences^(25,33)^, as illustrated in Figure 8. In chronic cases, these first two types of involvement cause fibrosis and ductal stenosis, which can be focal or diffuse. In the chronic phase, the findings of ischemic cholangiopathy may resemble those of primary sclerosing cholangitis. Therefore, the appropriate clinical context and direct evidence of hepatic artery thrombosis are key to the diagnostic reasoning^(17)^.

Obstruction of the hepatic arteries and arterioles can have various causes; those most commonly associated with ischemic cholangiopathy include thrombosis after liver transplantation, intra-arterial chemoembolization, iatrogenic vascular injury after gastrointestinal tract surgery, hereditary hemorrhagic telangiectasia, and polyarteritis nodosa^(33,34)^. Arterial thrombosis is the most common vascular complication after transplantation and usually occurs between 15 days and 6 months after the procedure, representing the second leading cause of graft loss, after rejection^(35)^.

## IATROGENIC CHOLANGIOPATHIES

Iatrogenic bile duct injury accounts for 95% of benign biliary strictures. Due to its high prevalence, it must be readily recognized, and imaging methods play a central role in its diagnosis. Approximately 80% of cases are diagnosed within the first year after surgery and can progress to liver cirrhosis if not treated properly^(36)^.

**Figure 9:**
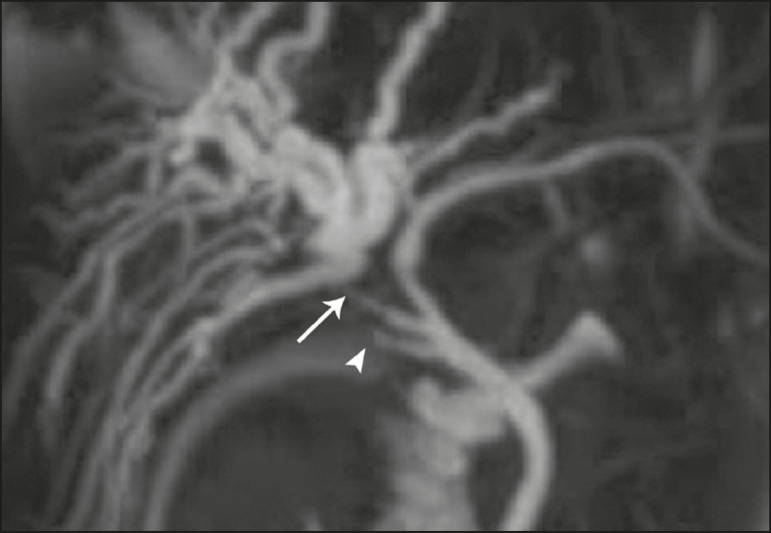
MRCP image of a patient in the postoperative period after cholecystectomy presenting with iatrogenic stenosis of the right hepatic duct (arrow), which has a low insertion into the common bile duct, near the cystic stump (arrowhead).

Surgical procedures that pose a risk of iatrogenic injury to the bile duct include those in which the hepatoduodenal ligament is manipulated, laparoscopic cholecystectomy being the most frequently associated^(36)^. There are several possible causes for this, the main one being the incorrect identification of the common bile duct^(36)^, which highlight the importance of preoperative MRCP to identify possible anatomical variations of the biliary tract. Another important tool recently made available is MRI with hepatobiliary-specific contrast agents. This imaging technique gives functional information of the bile duct, providing greater accuracy in the identification of anatomical variations-especially in patients without biliary dilatation-and in the characterization of postoperative biliary stenosis and fistulas^(10,37)^. The most common anatomical variations seen in iatrogenic cholangiopathies are as follows^(38)^: common bile duct running parallel to an aberrant duct; long cystic duct with low medial insertion; right aberrant duct draining into the left bile duct; and triple confluence of the intrahepatic bile duct, as shown in Figure 9. The initial treatment of choice is endoscopic or percutaneous bile duct dilatation; surgical reconstruction should be reserved for cases of treatment failure^(36)^.

## DIAGNOSTIC ALGORITHM

Before narrowing down the diagnostic possibilities to benign cholangiopathies, cholangiocarcinoma should be ruled out. To that end, a series of imaging findings that indicate malignancy must be analyzed^(26,28)^: long stenotic segments (> 12 mm); increased duct wall thickness (> 3.0 mm); hyperenhancement in relation to the liver parenchyma in the portal or equilibrium phase; parietal asymmetry; and luminal irregularity. Although the diagnostic accuracy of those findings is low when they are analyzed in isolation, it can reach 90% when they are analyzed together^(26,28)^.

The suspicion of a neoplasm is raised when there are elevated serum levels of tumor markers, CA 19-9 being the most widely used^(39)^. The CA 19-9 tumor marker is expressed by the pancreatobiliary epithelium, in benign and malignant conditions, as well as by several epithelial tumors at other sites^(40,41)^. In patients with cholestasis and without a history of primary sclerosing cholangitis, the proposed threshold is 90 U/mL, which has a sensitivity of 61% and a specificity of 95%^(40)^; in patients with cholestasis and a history of primary sclerosing cholangitis, it’s 129 U/mL, which has a sensitivity of 78.6% and a specificity of 98.5%. It is noteworthy that up to one third of patients with primary sclerosing cholangitis have CA 19-9 levels above (or even well above) the latter cutoff point^(27)^. Other causes of elevated CA 19-9 levels are cirrhosis and acute or chronic pancreatitis^(40)^.

**Figure 10:**
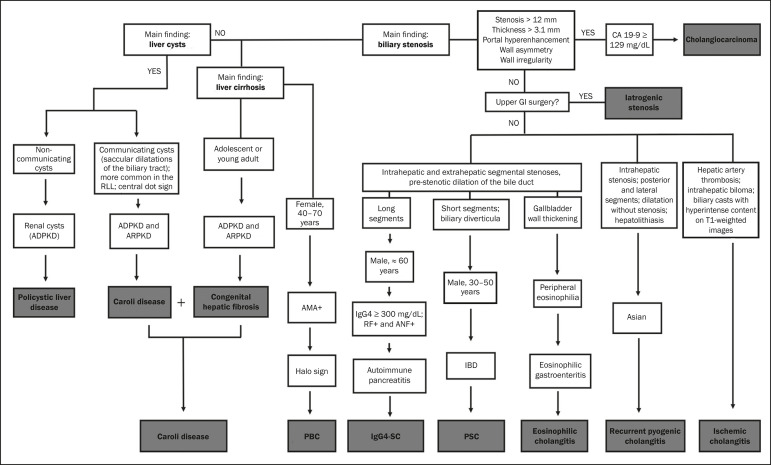
Diagnostic algorithm for non-neoplastic cholangiopathies. ADPKD, autosomal dominant polycystic kidney disease; ARPKD, autosomal recessive polycystic kidney disease; RLL, right liver lobe; AMA, antimitochondrial antibody; PBC, primary biliary cholangitis; CA, carbohydrate antigen; GI, gastrointestinal; GB, gallbladder; RF, rheumatoid factor; ANF, antinuclear factor; IgG4-SC, IgG4-related sclerosing cholangitis; IBD, inflammatory bowel disease; PSC, primary sclerosing cholangitis.

After ruling out cholangiocarcinoma, the appearance of the lesions on imaging should be considered in conjunction with their epidemiological and biochemical characteristics. To facilitate the diagnostic workup and shorten the time between the onset of the clinical picture and the introduction of the appropriate treatment, we have proposed an algorithmic approach (Figure 10).

## CONCLUSION

Non-neoplastic cholangiopathies present imaging findings, signs, and symptoms that are similar and can also resemble those of other conditions, such as post-traumatic cholangiopathy^(42)^ or even tumors of the biliary tract, in which case a histological study is necessary^(43)^. Only when the radiological, clinical, and epidemiological features of each of these entities are evaluated together we can narrow down the diagnostic possibilities and start treatment earlier.
